# Should Asymptomatic and Low-Risk Individuals be Tested for SARS-CoV-2?

**DOI:** 10.1007/s11606-020-06000-z

**Published:** 2020-06-24

**Authors:** Ashwini R. Sehgal

**Affiliations:** Center for Reducing Health Disparities, Case Western Reserve University, 2500 Metro Health Drive, Cleveland, OH USA

## INTRODUCTION

Because people with asymptomatic SARS-CoV-2 infections are an important source of transmission, widespread testing has the potential to identify and isolate such individuals, quarantine their contacts, and prevent further spread. However, testing for a condition in a group with low disease prevalence may lead to numerous false positive results. This study quantifies the tradeoff between reducing transmission by true positives and unnecessary isolation and quarantine due to false positives.

## METHODS

The impact of testing was modeled based on the following parameters: 1% prevalence of active infection, test sensitivity 90% and specificity 95%, reproduction number 0.9, and 5 contacts per case.^1–5^ Because performance of SARS-CoV-2 tests is not well established, the values that correspond to PCR-based tests for other respiratory viral infections were used.^2, 3^ The reproduction number *R* is the median value in the USA.^4^ Because individuals with positive test results will be instructed to isolate, *R* following a positive test was assumed to decrease to 0.1.^5^ The number of people subsequently infected by each infected case disease was calculated using the formula *R* + *R*^2^ + *R*^3^ + … = 1/(1−*R*)−1 when *R* < 1. For *R* = 0.9, this sum of 9.0 represents the number of new infections in a transmission chain and was applied to individuals with false negative results and to infected individuals who were not tested. The corresponding sum for a true positive case is 1.0, which assumes a reproduction number of 0.1 for the first transmission and 0.9 for subsequent transmissions.

## RESULTS

**Figure 1:**
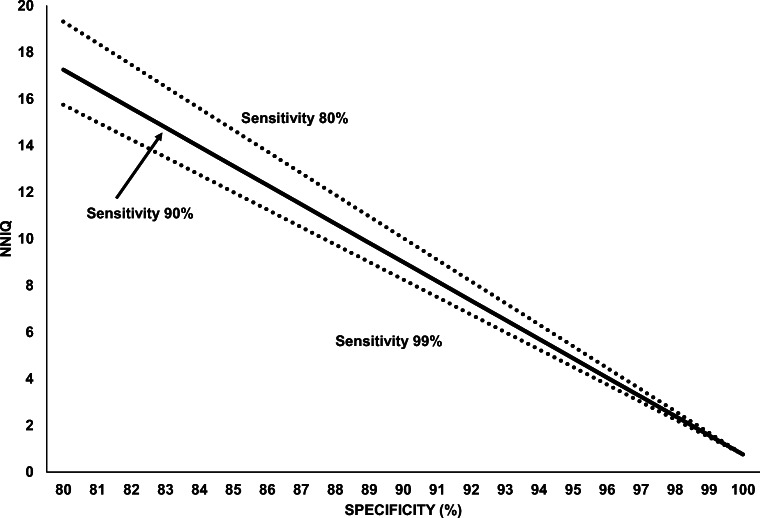
The number of people needed to isolate or quarantine (NNIQ) to prevent one infection as a function of test performance. From top to bottom, the three lines represent test sensitivity of 80%, 90%, and 99%.

In the base case, testing will result in 5.5 times more false positive than true positive results (Table 1). Testing will result in 72,000 fewer infections. Of the 351,000 people who will be isolated or quarantined, a substantial majority (297,000) will be isolated or quarantined because of false positive tests. With a higher prevalence of active infection, there will be 180,000 fewer infections, but a large number of people will still need to be isolated or quarantined. A highly specific test yields fewer false positives. About half (54,000) of the 113,400 people who must be isolated or quarantined will be because of true positive tests. A test that is both highly specific and sensitive will also greatly reduce the number of false negatives. At very high levels of test specificity, the number of people needed to isolate or quarantine (NNIQ) per infection prevented decreases and is minimally affected by variations in test sensitivity (Fig. 1).

**Table 1:** Simulated Results of One-Time Testing of 1 Million Asymptomatic and Low-Risk Individuals for SARS-CoV-2

	Base case	Higher prevalence of active infection	Better test specificity	Better test sensitivity and specificity
Model parameters	• Prevalence of active infection: 1% • Test sensitivity, specificity: 90%, 95% • Reproduction number: 0.9 (0.1 after positive test) • Contacts per case: 5	• Prevalence of active infection: 2.5% • Test sensitivity, specificity: 90%, 95% • Reproduction number: 0.9 (0.1 after positive test) • Contacts per case: 5	• Prevalence of active infection: 1% • Test sensitivity, specificity: 90%, 99% • Reproduction number: 0.9 (0.1 after positive test) • Contacts per case: 5	• Prevalence of active infection: 1% • Test sensitivity, specificity: 99%, 99% • Reproduction number: 0.9 (0.1 after positive test) • Contacts per case: 5
Results of testing	• True positive: 9000 (0.9%) • False positive: 49,500 (5.0%) • True negative: 940,500 (94.1%) • False negative: 1000 (0.1%)	• True positive: 22,500 (2.3%) • False positive: 48,750 (4.9%) • True negative: 926,250 (92.6%) • False negative: 2500 (0.3%)	• True positive: 9000 (0.9%) • False positive: 9900 (1.0%) • True negative: 980,100 (98.0%) • False negative: 1000 (0.1%)	• True positive: 9900 (1.0%) • False positive: 9900 (1.0%) • True negative: 980,100 (98.0%) • False negative: 100 (0.01%)
Transmission chain of infection if testing	• From 9000 true positives: 9000 new cases • From 1000 false negatives: 9000 new cases	• From 22,500 true positives: 22,500 new cases • From 2500 false negatives: 22,500 new cases	• From 9000 true positives: 9000 new cases • From 1000 false negatives: 9000 new cases	• From 9900 true positives: 9900 new cases • From 100 false negatives: 900 new cases
Transmission chain of infection if no testing	• From 10,000 infected individuals: 90,000 new cases	• From 25,000 infected individuals: 225,000 new cases	• From 10,000 infected individuals: 90,000 new cases	• From 10,000 infected individuals: 90,000 new cases
Isolation and quarantine due to testing	• Isolation: 58,500 positive test results • Quarantine: 292,500 contacts	• Isolation: 71,250 positive test results • Quarantine: 356,250 contacts	• Isolation: 18,900 positive test results • Quarantine: 94,500 contacts	• Isolation: 19,800 positive test results • Quarantine: 99,000 contacts
Overall effect of testing	• 72,000 fewer infections • 351,000 people isolated or quarantined • 4.9 number of people needed to isolate or quarantine (NNIQ) per infection prevented	• 180,000 fewer infections • 427,500 people isolated or quarantined • 2.4 NNIQ	• 72,000 fewer infections • 113,400 people isolated or quarantined • 1.6 NNIQ	• 79,200 fewer infections • 118,800 people isolated or quarantined • 1.5 NNIQ

## DISCUSSION

This analysis indicates that policy makers should avoid instituting mass testing of asymptomatic and low-risk individuals until a test with very high specificity becomes available. Determining test specificity will be challenging in the absence of a gold standard that definitively establishes the presence of absence of SARS-CoV-2. There are several possible approaches to clarify the magnitude of false positive results. A second test can be performed, preferably from a different manufacturer, to determine if it is negative. A serological test can be performed at a later date to determine if antibodies to SARS-CoV-2 develop as expected after infection. Individuals can also be followed over time to determine if they subsequently develop COVID-19 symptoms.

Several limitations must be considered in interpreting these results. The results do not apply to testing of symptomatic or high-risk groups. The actual values of model parameters have not been well established. The analyses assume that these parameters do not change over time. The number of contacts per infected individual (and therefore the number quarantined) may be higher or lower than the estimate used in these analyses. For example, the number of people who need to quarantine would be lower if the same individual is the contact of more than one infected person. Alternatively, much higher numbers of contacts, i.e., 10–30 per case, have been reported in some settings.^6^ As a result, it will be important to continue some social isolation restrictions to limit the number of people who must be quarantined. In addition, it is unclear whether contacts who have already recovered from COVID-19 or are known to have antibodies need to be quarantined. In contrast to more complex and less transparent models, these parameters and accompanying calculations can be easily modified for other scenarios and updated as the pandemic evolves.
